# Gellan Gum Is a Suitable Biomaterial for Manual and Bioprinted Setup of Long-Term Stable, Functional 3D-Adipose Tissue Models

**DOI:** 10.3390/gels8070420

**Published:** 2022-07-05

**Authors:** Franziska B. Albrecht, Vera Dolderer, Svenja Nellinger, Freia F. Schmidt, Petra J. Kluger

**Affiliations:** 1 Reutlingen Research Institute, Reutlingen University, 72762 Reutlingen, Germany; franziska.albrecht@reutlingen-university.de (F.B.A.); svenja.nellinger@reutlingen-university.de (S.N.); freia.schmidt@reutlingen-university.de (F.F.S.); 2 Faculty of Natural Science, University of Hohenheim, 72762 Reutlingen, Germany; 3 Faculty Science, Energy and Building Services, Esslingen University, 73728 Esslingen am Neckar, Germany; vera-dolderer@outlook.com; 4 Faculty of Applied Chemistry, Reutlingen University, 72762 Reutlingen, Germany

**Keywords:** hydrogels, 3D bioprinting, long-term stability, human primary ASCs, adipocytes

## Abstract

Due to its wide-ranging endocrine functions, adipose tissue influences the whole body’s metabolism. Engineering long-term stable and functional human adipose tissue is still challenging due to the limited availability of suitable biomaterials and adequate cell maturation. We used gellan gum (GG) to create manual and bioprinted adipose tissue models because of its similarities to the native extracellular matrix and its easily tunable properties. Gellan gum itself was neither toxic nor monocyte activating. The resulting hydrogels exhibited suitable viscoelastic properties for soft tissues and were stable for 98 days in vitro. Encapsulated human primary adipose-derived stem cells (ASCs) were adipogenically differentiated for 14 days and matured for an additional 84 days. Live-dead staining showed that encapsulated cells stayed viable until day 98, while intracellular lipid staining showed an increase over time and a differentiation rate of 76% between days 28 and 56. After 4 weeks of culture, adipocytes had a univacuolar morphology, expressed perilipin A, and secreted up to 73% more leptin. After bioprinting establishment, we demonstrated that the cells in printed hydrogels had high cell viability and exhibited an adipogenic phenotype and function. In summary, GG-based adipose tissue models show long-term stability and allow ASCs maturation into functional, univacuolar adipocytes.

## 1. Introduction

Adipose tissue plays a central role in lipid storage and metabolism [1] and exhibits a high endocrine function [2]. The main and characteristic cell type, mature adipocytes [3], is responsible for lipid metabolism and the production and release of adipokines. They are characterized by a round morphology with one big lipid-filled vacuole [2]. The precursors of mature adipocytes are adipose-derived stem cells (ASCs), which can be easily isolated from adipose tissue and expanded in vitro [4]. Using physical (e.g., stiffness of culture surrounding) and chemical (e.g., cell culture media supplements) cues, ASCs can be differentiated along the adipogenic lineage. Adipokines, secreted by cells of adipose tissue, influence the whole body’s metabolism, e.g., regulating blood pressure, amino acid metabolism, food intake, and immune reactions [5,6,7,8]. These wide-ranging effects also lead to the involvement of adipose tissue in many diseases and their pathogenesis [9,10]. 

Tissue models can be used in vitro for disease modeling, drug screening, basic research, and in vivo to replace, restore or repair tissue defects [11]. One strategy to develop such models in vitro is tissue engineering, which combines cells, scaffold materials, and soluble factors to create artificial tissues. For adipose tissue, in addition to scaffold-based models, models with mature adipocytes or spheroid-based models also find application in adipose tissue engineering [12]. This shows the wide range of possibilities for building sophisticated adipose tissue in vitro.

In addition to the cells, an essential part of each tissue is the extracellular matrix (ECM). Therefore, recreating the cell-surrounding matrix in vitro is one critical factor in developing artificial tissue models. The used scaffold material should mimic the characteristics of the native ECM. Commonly used ECM alternative for 3D models are hydrogels. They build a network with a high amount of water between the crosslinked polymer chains [13]. Due to the similarities to native glycosaminoglycans, hydrogels based on polysaccharides are often used for the in vitro setup of soft tissues [14]. As a linear microbial exopolysaccharide, GG possesses many favorable features such as biocompatibility, biodegradability, and a non-toxic nature. Further favorable properties are its high transparency, thermoresponsive features, flexible mechanical properties, ease of manufacturing and crosslinking, stability under physiological conditions, and low price [15,16,17]. This makes GG a promising material in tissue engineering and regenerative medicine, underlined by its widespread use in cartilage [18,19,20], bone [21,22,23], osteochondral [24] engineering, and spinal cord injury regeneration [25]. Furthermore, GG also applies to skin replacements comprising the subdermal part and the neovascularization in deep skin wounds, indicating its promising use in the field of adipose tissue [26,27]. Since 2D cultures lack a physiological micro-environment and animal models are complex and ethically controversial, in vitro 3D models are a good alternative. However, most studies use 2D cultures based on murine cell lines or primary cells that have provided important insights into biological processes such as browning [28] or beiging of adipocytes, studying tissue [29] or disease [30] mechanisms on a molecular level. However, there is evidence that shifting in vitro models into the third dimension leads to more reliable results [31,32] as they mimic the in vivo microenvironment more closely.

Only limited studies were found using a human 3D adipose tissue model as spheroids or microfluidic system [33,34,35,36,37] to mimic the native situation in more detail. Despite all studies creating important data for understanding mechanisms in adipose tissue, they rely on not fully matured ASCs, xenogenic cells, or have only been evaluated over a short period. The distinct features of differentiated ASCs compared to mature adipocytes [38] outline the importance of tissue models based on fully matured adipocytes. In addition, it is known that the transferability of animal-based models to humans is limited [39]. Long-term stability is an important aspect of establishing an in vivo-like adipose tissue model as ASCs need time to develop into mature adipocytes and exhibit characteristic functions, which in turn are essential for whole tissue function. 

In the present study, we demonstrated the development of long-term stable adipose tissue models based on 3D GG hydrogels. The mature state and functionality of the differentiated ASCs (diffASCs) were examined by visualization of accumulated lipids, characteristic protein expression, and secretome analysis. Further, additive manufacturing was established using extrusion-based bioprinting. The reproducible cell-containing lattice structures built this way were in no way inferior to the manually created models in terms of viability and functionality.

## 2. Results

### 2.1. Gellan Gum Hydrogels Exhibit Soft Tissue Properties and Long-Term Stability

**Figure 1:**
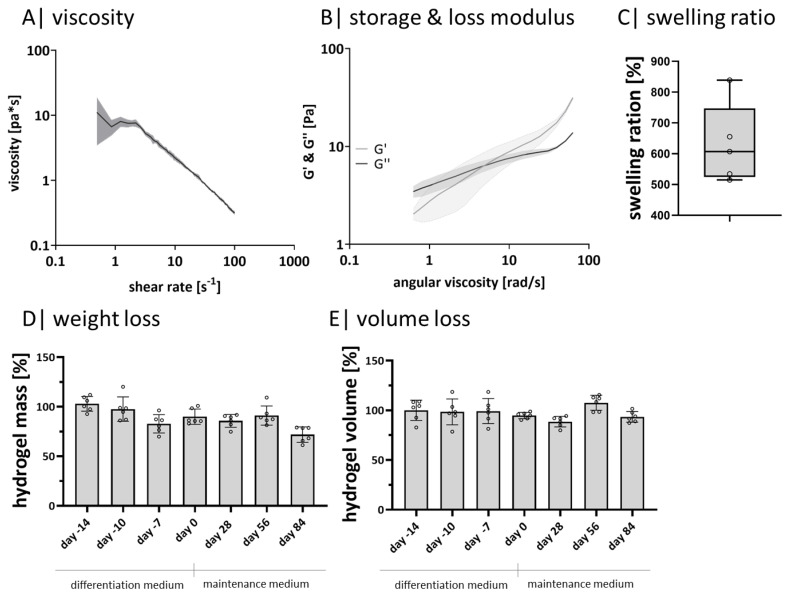
Hydrogel characteristics: (**A**) rheological measurement of the viscosity and (**B**) of the storage and loss modulus with a rotational viscometer, n = 3; (**C**) calculated swelling ratio of acellular, crosslinked hydrogels after 24 h, n = 6; (**D**) measured weight maintenance of crosslinked acellular hydrogels over 98 days, n = 6; (**E**) determined normalized volume change of crosslinked acellular hydrogels over 98 days, n = 6. ◦ sample values.

Since adipose tissue is classified as soft tissue, potential matrix materials must exhibit comparable physical characteristics. Therefore, the material properties viscosity, storage modulus (G’), loss modulus (G”), and swelling ratio were determined to verify acellular 1% GG hydrogels’ characteristics (Figure 1). During shear thinning, the viscosity decreases (Figure 1A), and by increasing the angular viscosity, G ‘and G” intersect (Figure 1B). With increased angular viscosity, G’ is higher than G”. The average swelling ratio is 630% (Figure 1C). Long-term stability of GG hydrogels was observed for 98 days by weight and volume progression (Figure 1D,E). The normalized weight (Figure 1D) and volume (Figure 1E) maintenance start on day −14 and end on day 84. Each value is normalized to the starting point of day −14. Both courses show slight but not significant fluctuations.

### 2.2. Gellan Gum Hydrogels Do Not Activate Monocytes or Induce Cytotoxic Reactions

**Figure 2:**
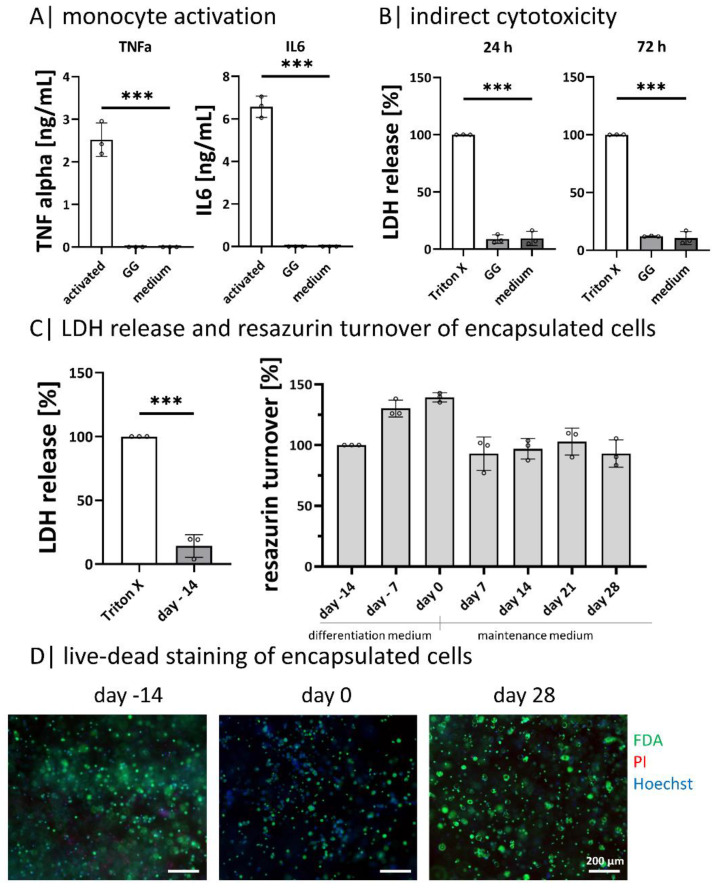
Cell-material interaction: (**A**) tracking of monocyte activation by the secretion of TNFα and IL6 after incubation in hydrogel supernatant, n = 3; (**B**) indirect cytotoxicity testing according to DIN EN ISO 10993-5 after 24 h and 72 h, n = 3; (**C**) diffASC resazurin turnover in GG-hydrogels over 42 days and LDH release after 24 h post-ASC encapsulation, n = 4; (**D**) live-dead staining of diffASC GG-hydrogels on days −14, 0, and 28, viable cells in green, dead cells in red, and nuclei in blue. Scale bar 100 µM, n = 5. ◦ sample values, *** *p* ≤ 0.001.

Metabolic activity and viability tests were performed to exclude cell-affecting effects of the exopolysaccharide GG on ASCs (Figure 2). Monocyte activation was tested to prove if GG activates monocytes due to residual pyrogens (Figure 2A). The GG-leachate did not result in an undesired activation of the monocytes since only the positive control activated the immune cells, where a significantly higher TNFα and IL6 secretion is found. It was not possible to detect cytokine release neither for the negative control nor for the samples incubated with GG-hydrogel supernatant (Figure 2A). Metabolic activity was analyzed by resazurin conversion, cell death by LDH release, and the overall cell viability by live-dead staining (Figure 2B–D).

The amount of released LDH was determined by indirect cytotoxicity testing after incubating ASCs with GG-hydrogel leachate after 24 h and 72 h. The Triton-X treated positive control was set as a reference point. For both time points, the amount of released LDH in GG-hydrogel leachate and the negative control (fresh cell culture medium) was significantly lower (Figure 2B). As the process of apoptosis is a part of the normal cell turnover and development, LDH is released in small amounts in the negative control as well as in the samples [40]. In direct cell contact, cells were not impaired by GG hydrogels. After encapsulation, the LDH release was significantly lower than the control (Triton-X treated). This is in line with the resazurin turnover during the culture, which increased to a higher level at day 0 (end of differentiation) and decreased with light fluctuations until day 28 (Figure 2C). Live-dead staining shows the encapsulated ASCs on days −14, 0, and 28. A few dead cells (in red) are visible on all days, while the majority is viable (in green) with a blue nucleus (Figure 2D). The viability staining on days 56 and 84 complete the course as there are no or only few dead cells visible (provided as Supplementary Figure S1). 

### 2.3. Gellan Gum Hydrogels Enable Adipogenic Differentiation and the Setup of Functional Adipose Tissue Models for 98 Days

**Figure 3:**
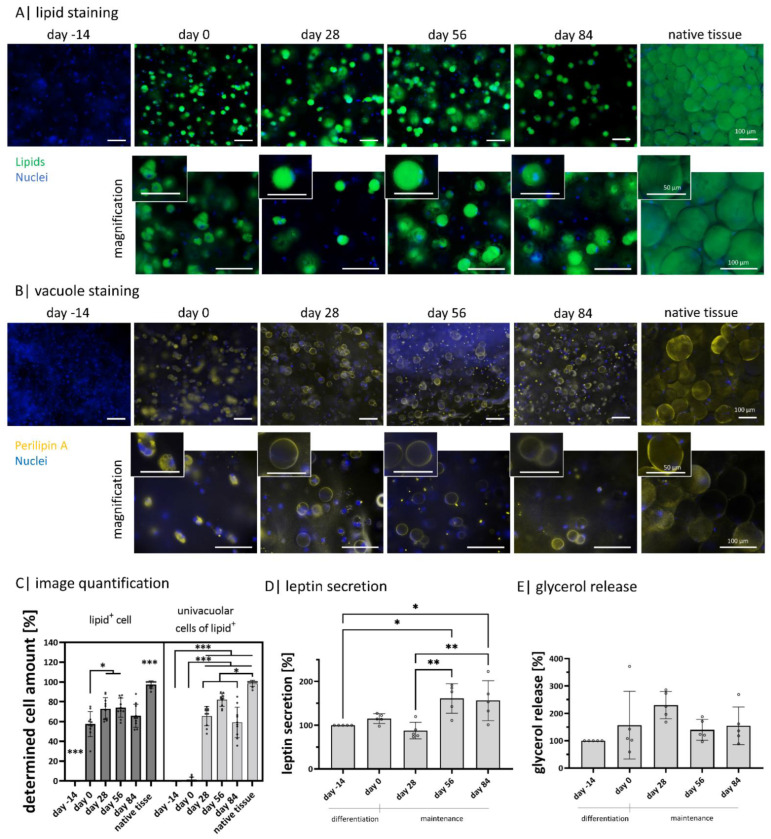
Intracellular lipids and adipokine secretion within Gellan gum hydrogels: (**A**) visualization of intracellular lipids of encapsulated diffASCs for 98 days and explanted lobuli after 24 h. Stained with BODIPY, lipids in green, nuclei in blue. Scale bar 100 µM, n = 5; (**B**) visualization of perilipin A as an intact lipid vacuole membrane protein in encapsulated diffASCs for 98 days and explanted lobuli after 24 h. Stained with anti-perilipin A antibody in yellow, nuclei in blue. Scale bar 100 µM, n = 5; (**C**) determined cell number expressing lipids (lipid^+^) and an univacuolar morphology (percentage of univacuolar cells to lipid^+^ cells), both increasing with increasing culture time; (**D**) normalized adipokine secretion (leptin) of diffASCs in GG-hydrogels for 98 days, n = 5; (**E**) normalized glycerol release of encapsulated diffASCs in GG-hydrogels for 98 days, n = 5. ◦ sample values, * *p* ≤ 0.05; ** *p* ≤ 0.01; *** *p* ≤ 0.001.

Mature adipocytes exhibit a characteristic round lipid-filled vacuole covered by perilipin A, among other perilipins [41]. This is seen for native adipose tissue in the form of explanted lobuli in Figure 3. As expected, they exhibit an univacuolar morphology with a comparably small nucleus next to the lipid-filled vacuole. Further, the visible cells express perilipin A on the vacuole membrane as a continuous ring. In order to show if the functionality of encapsulated diffASCs in long-term culture is comparable to native adipose tissue, these characteristic properties were evaluated at five time points: days −14 (induction of differentiation), 0 (end differentiation, start maintenance), 28, 56, and 84 (Figure 3) and compared to native adipose tissue. On day −14, the ASCs did not show any intracellular lipids. During adipogenic differentiation and maintenance, intracellular lipids (green) increased significantly. Their accumulation started after induction via media with multiple smaller vacuoles (until day 28), and afterward, they fused to one big vacuole. The differentiated cells appear to have a functional maximum between days 28 and 56. Most cells appear univacuolar, with the nucleus located at the membrane. It seems there is a slight decrease in stored lipids and the number of differentiated cells drops after day 56 (Figure 3A,C). For perilipin A, the functional maximum is between days 28 and 56, where 73% and 74.1% lipid-positive cells could be detected (Figure 3B,C). At day −14, no perilipin A is detected, while its appearance increases during adipogenesis. From day 28, perilipin A is expressed as a continuous ring around the vacuole in most cells. This staining supports the assumption that there is a decline between days 56 and 84, which is confirmed by the decreasing number of univacuolar cells (Figure 3B,C). A further important function of adipocytes is the secretion of adipokines, such as leptin. The normalized leptin secretion fluctuates (non-significant) between days −14, 0, and 28 and is significantly lower than on days 56 and 84. Indicating a maximum cellular function around day 56 (Figure 3D). Next to the primary function of adipocytes to store lipids, they can degrade and release these lipids. A side product of the degradation of lipids is glycerol which can be measured in a cell culture medium. The released glycerol (Figure 3E) has its significant peak on day 28. Nevertheless, the levels on days 56 and 84 are higher than the initial levels on days −14 and 0. 

### 2.4. Gellan Gum Can Be Bioprinted 

The development of a bioink with high print fidelity and high cell viability is challenging [42]. For the purpose of this study, the composition of the GG hydrogel was adjusted to be printable. Therefore, the number of divalent ions was increased.

**Figure 4:**
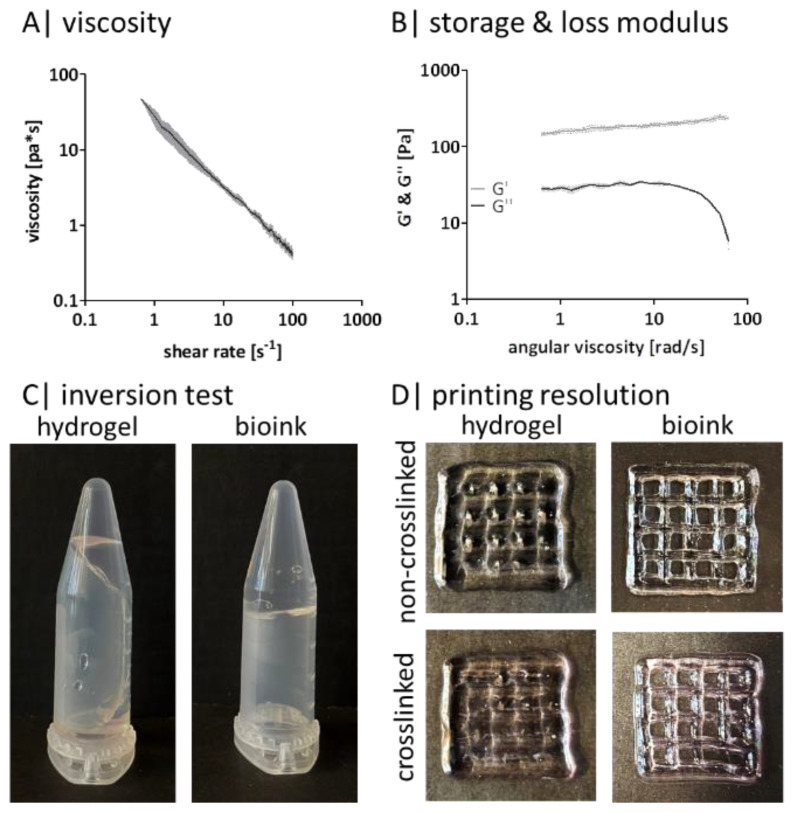
Gellan gum bioink characteristics: rheological measurement of (**A**) the viscosity and (**B**) of the storage and loss modulus with a rotational viscometer, n = 3; (**C**) representative image of an inversion test of (a) the hydrogel composition and (b) the bioink composition; (**D**) representative printing resolution with a 20 × 20 × 2 mm grid (20% infill, 410 µm nozzle) of left hydrogel composition and right bioink composition before (upper picture) and after crosslinking (lower picture).

The addition of divalent ions changes the viscosity, storage, and loss modulus of the adjusted bioink (Figure 4). The viscosity decreases with increasing shear rates (Figure 4A). Even with increasing angular viscosity, G’ stays constantly above G” (Figure 4B). As the inversion test shows, the hydrogel composition exhibits less stability and flows along the tube edge. In contrast, the bioink composition is stable, and no flow down is visible (Figure 4C). This behavior is also reflected in the printing resolution tests (Figure 4D). The printed grid appears blurred when using the hydrogel—before and after crosslinking. The individual grid holes are visible as slight cavities, and the outer edge seems to be washed out. Using the bioink, the contours of the grid structure are clearly visible, and the individual holes can be perceived as small squares within the grid before and after crosslinking.

### 2.5. Bioprinted Gellan Gum Hydrogels Are Not Cytotoxic and Enable Similar Adipogenic Differentiation and Functionality as Manually Produced Gellan Gum Hydrogels 

**Figure 5:**
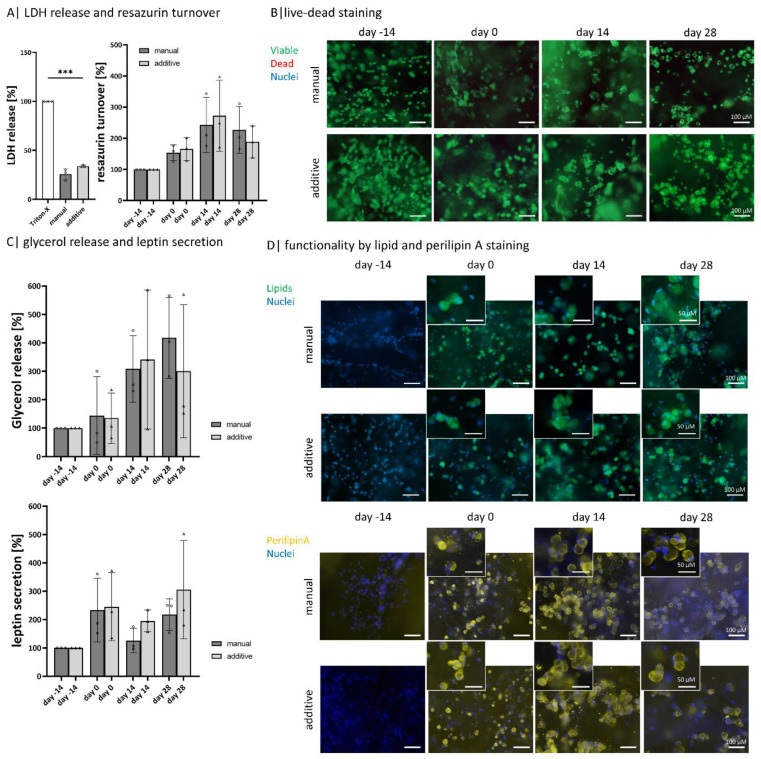
Comparison of bioprinted and manual models: Compared analysis of encapsulated diffASCs days −14, 0, 14, and 28 in (**A**) live-dead staining, viable cells ins green, dead cells in red, scale bar 100 µm (**B**) quantitative LDH release and resazurin turnover, n = 3, *** *p* ≤ 0.001; (**C**) normalized glycerol release and leptin secretion, n = 3; ◦, ^∆^ sample values, (**D**) staining of intracellular lipids (in green, nuclei in blue) and perilipin A (in yellow, nuclei in blue) as functionality marker, scale bar 100 µm for magnification 50 µm.

It is known that bioprinting can affect cell viability [43]. Therefore, it is important to determine the influence on cell behavior caused by the bioprinting process in terms of viability and functionality (Figure 5). 

The overall viability of encapsulated ASCs within the bioink composition hydrogels on days −14, 0, 14, and 28 is high with both manufacturing methods, as nearly no dead cell is visible in the live-dead staining (Figure 5A). Nevertheless, the released LDH amount indicates 25% (for manual models) and 30% (for additive models) of dead cells. The metabolic activity, determined by resazurin turnover, increases until day 14 and decreases again until day 28 for both model types (Figure 5B). By visualizing the intracellular lipid content with BodiPY, the same steps in adipogenic differentiation can be observed for both manufacturing methods: ASCs have no lipids on day −14 and exhibit a multivacuolar morphology on day 0, which in the course of maintenance becomes an univacuolar morphology (Figure 5C). These changes are also demonstrated by visualizing the outer membrane of the lipid vacuole with perilipin A staining, equally expressed in both model types (Figure 5D).

Both manufacturing methods show a similar tendency if the glycerol releases are considered. There is an increase until day 14, which decreases for additive models until day 28 but slightly increases for manual ones (Figure 5E). The amount of leptin secreted increases for both types of models. On days 0 and 28 there is an increase, while on day 14, there is a slight decrease compared to days 0 and 28. The additive models show a stronger increase than the manual models.

## 3. Discussion

To reach a functional and long-term stable, in vivo close adipose tissue model, we developed a GG-based hydrogel containing primary human ASCs, which successfully differentiated into adipogenic phenotypes. 

In addition to cell selection, the material is a critical factor in the setup of a tissue model. It is advisable to use a material similar to native ECM. Gellan gum was chosen because it is described as similar to glycosaminoglycans [41] and is therefore ECM-like. Furthermore, multiple in vitro and in vivo studies demonstrated that GG is a non-toxic, biocompatible polysaccharide [16,44,45,46,47,48].

For successful engineering of tissue constructs, it is not only the chemical origin of a hydrogel material that is decisive but also the stability during cell culture. Therefore, we tested acellular GG hydrogel stability before cell encapsulation. The rheologically determined viscosity, storage, and loss modulus demonstrated that GG hydrogels exhibit the expected features of a soft hydrogel suitable for adipose tissue. Based on the swelling ratio after 24 h, it can be concluded that the material possesses a high water binding capacity, which is in line with the literature [49]. The slight changes in weight and volume might be based on minor, non-significant material washouts, also observed by others [50]. Summarized, 1% GG-hydrogels exhibit long-term stability under cell culture conditions up to day 98, making the hydrogels a promising candidate for cell encapsulation.

In general, the results show that GG does not exhibit any cytotoxicity—neither in indirect nor direct (as encapsulated cells) contact, which is in line with the literature [51]. The low LDH release shows this compared to the control (Triton-X treated), the resazurin turnover, and the life-dead staining on days −14, 0, and 28. Further, GG leachates did not activate the monocytes, as no TNFα and IL6 were released. This suggests that there are insufficient pyrogen residues within the GG material for the activation of monocytes [52,53].

As the stiffness of the surrounding matrix strongly affects the differentiation of the stem cells, it is advisable to use a soft matrix for adipose tissue [54]. It was shown that a soft matrix and an initial round cell morphology without focal adhesion [55] promote adipogenesis, while a stiffer matrix and elongated cell morphology promote osteogenesis [56]. The successful adipogenic differentiation of encapsulated ASC is confirmed by intracellular lipid staining and perilipin A staining. Starting on day −14, no lipids or perilipin A was visible. At the end of differentiation (day 0), multiple smaller vacuoles were arranged around the cell nucleus, each expressing individual perilipin A. These multiple vacuoles fused to one bigger vacuole during maintenance, indicating adipocyte maturation [57]. If the staining of the native tissue is considered, the cell morphology and perilipin A expression of the diffASCs in the hydrogel are very similar. One lipid-filled vacuole next to the cell nucleus or the continuous perilipin A expression as a ring, respectively. Even if the diameter is smaller, this may indicate both the maturation duration and the cells’ health status. Very large cell volumes are increasingly found in pathophysiological states (hypertrophy) [58]. As nearly all visible cells showed up a characteristic round morphology and a vacuole next to the nucleus, it can be concluded that a high differentiation rate was reached. Slight signs of dedifferentiation are noticeable from day 56 to 84. The vacuoles seem to decrease in size, and fewer cells exhibit intracellular lipids and perilipin A. Dedifferentiation can be caused by multiple physiological (e.g., tissue regeneration) and pathophysiological factors [59]. The probable reason is the duration of the culture in which the cells did not experience enough stimuli after that long time.

These findings are supported by the quantitative glycerol release and leptin secretion. For the glycerol release, a peak was found on day 28. Higher glycerol levels indicate cleaved lipids, but the presumably larger amount is based on an increased lipid turnover [60]. It might be assumed that cells around day 28 primarily focus on lipid synthesis instead of characteristic adipocyte functions like adipokine secretion. On days 56 and 84, a significant increase was detected in leptin secretion, indicating that the matured cells fulfilled characteristic functions [57]. In sum, all suggest that GG is a suitable material for encapsulation, adipogenic differentiation, and maintenance of human primary ASCs. This was also demonstrated by another study which has shown the development of adipose-like microtissues based on GG [61]. Like us, they have shown successful adipogenic differentiation of encapsulated ASCs in different GG concentrations. However, the type of application target differs, as in this case, GG is intended as a vehicle for injections. Only a few studies were found focusing on the use of longer maintained ASCs or mature adipocytes. The culture of miniaturized mature adipocyte gels in fluidic systems shows promising results concerning functionality and flexibility in vitro [35]. Nevertheless, there is optimization potential in the long-term application and the accessibility and storage of the used cells. Although spheroids are shown to be a suitable model for the differentiation and maturation of functional, univacuolar adipocytes [62], the use of animal cells allows only limited conclusions to be drawn about human cells.

A remarkable similarity between cell culture models and the target in vivo situation is crucial for the comparability and thus reliability of the results produced in vitro. We have combined the current requirements for in vitro models [63] to develop an adipose tissue model that is structurally and functionally more similar to the in vivo situation. This study has shown that GG hydrogels are a promising approach to create viable, functional, and long-term stable adipose tissue models with fully matured adipocytes. Encapsulated cells accumulated intracellular lipids, expressed cell-characteristic proteins, released adipokines, and exhibited a roundish, univacuolar cell morphology. In summary, diffASCs within the models appeared as mature adipocytes as they are found in vivo.

Bioprinting is often used as a manufacturing method as it offers the possibility of spatially controlled deposition of cell-containing bioinks. This allows models to be built with defined pores, circumventing the diffusion-limited supply of larger constructs known to arise in bulk models. Additive manufacturing allows the reproductive generation of high numbers of models, which in turn can be considered for use as a test system. Concerning adipose tissue, a future aim is to bioprint large-volume constructs to replace missing subcutaneous adipose tissue patient-specifically [64]. Nevertheless, it is known that the development of a bioink is one of the most complex parts of bioprinting [43]. This is due to the multiple possibilities to adjust the bioink itself and the parameters during bioprinting. Additionally, other researchers have already shown that bioprinting of defined structures with high cell density is beneficial for adipogenesis and maturation [65]. In our case for GG, the ions stabilize the previously formed double helix and increase the viscosity, whereby the effect of divalent ions is stronger [17] than the one of monovalent ions, which in turn help to stabilize the gels [66]. Our rheological data prove that the additionally added ions increase the viscosity and, therefore, enhance G’. Furthermore, G’ is immediately greater than G”. Due to the higher ion concentration in the bioink compared to the hydrogel composition, it can be assumed that a higher crosslinking density is present within the bioink. The inversion test and the bioprinting resolution further confirm that the added ions visibly increased stability at room temperature and print fidelity. In summary, a successful adjustment was achieved to enhance the processing and bioprinting features by the careful addition of mono- and divalent ions in the crosslinking medium.

Bioprinting can influence cell viability; hence, we directly compared the two manufacturing methods (manual and additive setup) to exclude negative influences [43]. Because the cells in the manual models had stable characteristics after day 28, the bioprinted constructs were examined up to this point. No significant differences in cell viability or functionality could be found—particularly for the stainings. There are minor deviations within the quantitative methods. The values for LDH release and resazurin turnover are slightly higher for additive models. This might be explained by the greater surface area of the models exposed to the printing nozzle and the cell culture media. Meaning that the cells, on the one hand, have a greater amount exposed to the printing nozzle but, on the other hand, a greater surface for resazurin access. The surface enlargement within the additive models could also explain the higher leptin amount and the lower glycerol levels within the additive models. As the cell culture media and especially the nutrients and differentiation factors can directly flow through the printed grid holes, the nutrient diffusion to the cells is quicker than in the manual cylinder model. The same holds for the release, respectively, for their distribution within the supernatant. Accelerated access to nutrients and differentiation factors can indicate the more pronounced adipogenic character.

Since the co-culture of endothelial cells and stem cells positively influences cell maturation and model development, further attempts will be made to investigate the co-culture of ASCs and endothelial cells [67,68].

## 4. Conclusions

This work showed that the polysaccharide GG is a promising material for adipose tissue engineering. Using 1%, GG enabled the setup of non-toxic, non-monocyte activating 98 days of long-term stable hydrogels. Furthermore, it facilitated the culture of viable human primary ASCs for 98 days, as well as successful adipogenic differentiation. During maintenance, encapsulated cells stored significant amounts of intracellular lipids exhibited a univacuolar morphology, and secreted adipokines, demonstrating its in vivo-close cell state and function. In addition, we established the extrusion-based bioprinting of pre-crosslinked GG and were able to exclude influences on cell viability or functionality that might arise from the process over 42 days.

## 5. Materials and Methods

### 5.1. Hydrogel Formulation

The hydrogel composition (used for hydrogel characteristics, cell-material interaction, and cellular hydrogels) contained 100 mg GG (Gelzan, Sigma Aldrich, Taufkirchen, Germany) in 9 mL double distilled water and 1 mL mesenchymal stem cell growth medium (MSCGMx, PeloBiotech). Additionally, the bioink composition (used for bioprinting experiments) contained 100 mg GG in 7.5 mL double distilled water, 1.5 mL MSCGMx, and 1 mL PBS+.

For both, GG was dissolved in double distilled water in the microwave by heating the liquid several times, stirring at a vortexer and heating again until a clear solution was obtained. Afterward, the solution was tempered at 37 °C, and the additional liquids (MSCGMx or MSCGMx and PBS+ with or without cells) were carefully added by pipetting up and down while stirring simultaneously. A measure of 100 µL of the resulting hydrogel-like solution were transferred into a mold (8 mm diameter, 3 mm height) within a 24-well plate (Greiner BioOne) using a 1 mL syringe (B.Braun). Finally, for crosslinking the unstable hydrogel-like solution, the molds were rinsed with 750 µL cell culture medium and placed into an incubator (Thermo Fisher Scientific) under standard conditions (37 °C and 5% CO_2_).

### 5.2. Hydrogel Analysis

Media exchange of acellular hydrogels was performed three times per week for two weeks and then for 12 weeks with two changes per week.

For determining the swelling ratio (1), acellular hydrogels were weighed after 24 h (wet) incubation, dried for 48 h at 60 °C (dry) in an oven (Memmert AG, Schwabach, Germany), and weighed again.

(1) $$\frac{\mathrm{weight}\;\left(\mathrm{wet}\right)-\mathrm{weight}\;\left(\mathrm{dry}\right)}{\mathrm{weight}\;\left(\mathrm{dry}\right)}\times 100$$

The normalized weight (2) was calculated by weighing them after removing all excess liquid.

(2) $$\frac{100\times \mathrm{sample}\;\mathrm{weight}}{\mathrm{average}\;\mathrm{weight}\;\mathrm{after}\;24\;h}$$

For volume determination, samples were taken, and the diameter and height were measured with a digital caliper (Alpha Tools). The volume was calculated according to (3) and the normalized volume according to (4).

(3) $$2\times \pi \times \mathrm{height}\times \frac{\mathrm{diameter}}{2}$$

(4) $$\frac{100\times \mathrm{sample}\;\mathrm{volume}}{\mathrm{average}\;\mathrm{volume}\;\mathrm{after}\;24\;h}$$

### 5.3. Rheological Analysis

Shear-thinning and frequency sweep were measured with a Rheotest RN4 (Rheotest). A plate-plate setup with a gap distance of 100 µM and a plate diameter of 35 mm at 22 °C was used for both. For shear-thinning, the share rate was constantly increased from 0 to 100 s^−1^ in 300 s (33 s^−1^ increment). For the frequency sweep, the linear viscoelastic region was determined at a constant amplitude of 25 Pa. Subsequently, the samples were oscillatory loaded with an increasing frequency from 0–10 Hz.

### 5.4. Cell and Lobuli Isolation

The source material for both isolations was human adipose tissue (Charlottenhaus Clinic, Stuttgart, Germany) from surgical skin reduction. For lobuli isolation, the adipose tissue was held carefully with forceps and cut along the connective tissue between the lobules with scissors. Care has been taken not to damage the individual cells. The individual lobuli were cultured for 24 h in 750 µL Adipocyte Maintenance Medium-1 (amsbio).

For ASC isolation, unused tissue parts such as blood vessels, connective or sclerosed tissue, and the epidermal and dermal skin layers were removed with scissors. Adipose tissue was cut into small fragments (~5 mm^2^) and digested using 100 U/mL collagenase (Serva) in Dulbecco’s Modified Eagle Medium (DMEM, Pan Biotech, Aidenbach, Germany) with 1% bovine serum albumin (Biomol, Hamburg, Germany) for 3 h at 37 °C under mild agitation. The suspension was passed through a 500 µm and a 200 µm mesh. As a result, a three-layered suspension was gained. The upper two contained lipids and were discarded. The lower aqueous phase was centrifuged, the supernatant was discarded, and the pellet was resuspended in erythrocyte lysis buffer (155 mM ammonium chloride, 10 mM potassium hydrogen carbonate, 0.1 mM ethylenediaminetetraacetic acid, EDTA) and incubated for 10 min at RT. Another centrifugation step followed, after which the suspension was passed through a 70 µM sieve (Greiner BioOne). Centrifuged ASCs were resuspended and seeded in MSCGMx with an initial density of 40,000 per cm^2^. Passaging took place at a confluence of 80–90%. ASCs were used up to passage 4.

### 5.5. ASC-Containing Hydrogels and Maintenance

For cellular 1% GG hydrogels, ASCs were trypsinized (0.05% trypsin-EDTA, Gibco in Versene, Lonza), and cell density was adjusted to 3 × 10^6^ cells per 100 µL MSCGMx. 100 µL cell suspension was mixed with 900 µL GG-solution by pipetting up and down while stirring simultaneously. Hydrogels were built up as described in Section 5.1. For cell culture, hydrogels were rinsed with 750 µL prewarmed DMEM with fetal calf serum (FCS, PanBiotech; DMEM-FCS, 10% FCS, 1% P/S, and 870 g/L glutamine) and placed into the incubator. After 24 h, the media was changed to DMEM-Diff (DMEM-FCS + 500 µM 3-Isobutyl-1-methyl-xanthine, 100 µM indomethacin, 1 µg/mL insulin, and 1 µM dexamethasone) for 2 weeks with three half media exchange weekly. For the next 12 weeks, half of the media was exchanged twice a week with DMEM-main (DMEM + 3% FCS + 4 µM biotin, 8 µm pantothenate, 1 µg/mL insulin, and 1 µM dexamethasone).

### 5.6. Bioprinting

For bioprinting and the comparison of manufacturing methods, all hydrogels were set up with an increased concentration of divalent ions. 3 × 10^6^ ASCs were resuspended in 150 µL MSCGMx with 100 µL PBS+ to achieve 1 mL cellular bioink. Manual bioink hydrogels were, except for the composition, fabricated as in 5.6. The ink was transferred into a 3 mL cartridge (cellink) for bioprinting. The bioprinted models were manufactured as a 10 × 10 × 2 mm grid with 20% infill density, 410 µm nozzle, print head 30 °C, print bed 20 °C, 20 mm/s velocity, and 10 kPa pressure. Cell culture conditions were the same as described in 2.5.

### 5.7. Monocyte Activation

Monomac 6 (ACC 124, German Collection of Microorganisms and Cell Cultures) were seeded with a 5 × 10^5^ cell density in a 24-well plate for 48 h in 1 mL (Roswell Park Memorial Institute medium with 1% P/S and 10% FCS). Acellular hydrogels were produced as in Hydrogel analysis and cultured for 24 h in 1 mL testing medium (DMEM without phenol red PanBiotech and 1% P/S). Monocytes were activated with lipopolysaccharides (1 µg/mL) and phorbol-12-myristate-13-acetate (12.3 ng/mL) as a positive control. The negative control was treated with fresh cell culture medium. After 24 h incubation, the sample and control media were collected, and secreted concentration of tumor necrosis factor-α (TNFα) and interleukin 6 (IL6) was determined via an enzyme-linked immunosorbent assay (ELISA).

### 5.8. Indirect Cytotoxicity

Testing of indirect cytotoxicity was based on DIN EN ISO 10993-5. Briefly, acellular hydrogels were fabricated as described in Hydrogel analysis and cultured for 24 h in 1 mL testing medium (as in 2.7). In parallel, 2 × 10^4^ ASCs per cm^2^ were seeded in a 24-well plate with MSCGMx with 1% P/S. As a positive control, 0.1% Triton-X (Sigma Aldrich, Taufkirchen, Germany) was added to the testing medium, and as a negative control testing medium only was incubated. The next day, 3% FCS was added to all testing media and given to the previously seeded cells. Supernatant samples were taken after 24 h and 72 h and analyzed for released LDH.

### 5.9. ELISA Antigen Determination

Three different products of interest were quantified via a sandwich ELISA: IL6 (a) and TNFα (b) as pro-inflammatory cytokines and leptin (c) (PeproTech, Germany) as an adipokine. ELISAs were carried out precisely according to manufacturer protocol. Samples a & b were diluted 1:1, and c was used undiluted. For color development, 100 µL tetramethylbenzidine (biozol) was added and incubated for 15 min. The reaction was stopped by adding 100 µL 1 M hydrochloric acid. Absorption measurement took place at a wavelength of 450 nm with a reference at 620 nm with a SpectraMax i3 (Molecular Devices).

### 5.10. Lactate Dehydrogenase Assay

For LDH determination (Takara Bio, Shiga, Japan), 50 µL of cell culture supernatant and 50 µL LDH reagent (1:50 catalysator:dye) were mixed in a 96-well plate. The plate was incubated for 30 min in the dark and measured at a wavelength of 490 nm and a reference set at 680 nm.

### 5.11. Resazurin Turnover

At 24 h before the measurement, a resazurin working solution (appropriate cell culture medium without phenol red and 0.11 µg/mL resazurin (Sigma Aldrich)) was given to the cells. A measure of 100 µL triplicates were pipetted into a 96-well plate, and the absorbance was measured at 570 nm with a reference at 600 nm.

### 5.12. Live-Dead Staining

For fluorescein diacetate (FDA) propidium iodide (PI) staining, gels were incubated at 37 °C for 15 min in staining solution (FDA: 10 mg/mL, Sigma Aldrich; PI: 5 mg/mL, Sigma Aldrich; Hoechst 33,342: 1 µg/mL, Cell Signaling in PBS+) and were washed twice with PBS+ for 15 min.

According to the manufacturer’s instructions, the second viability staining (for additive and manual models) was performed: 1 mL PBS+ was supplemented with 0.5 µL Calcein, 2 µL ethidium homodimer, and 1 µL Hoechst 33,342 and incubated for 60 min. Microscopy took place with an Axio Observer microscope and an Axiocam 305 color camera using ZENblue software (all Carl Zeiss).

### 5.13. Glycerol Measurement

According to the manufacturers’ instructions, an assay kit (Randox Laboratories) measurement was performed. First, the 24 h cell culture supernatants were diluted in buffer (1:1), and a standard series in buffer (0–100 ng/mL) was prepared. A measure of 100 µL duplicates were pipetted into a 96-well plate, mixed with 100 µL glycerol assay reagent, and after a 10 min incubation, the measurement at 520 nm took place.

### 5.14. Staining of Intracellular Lipids

Intracellular lipid content was visualized by staining with the lipophilic dye BodiPY 493/503 (Biomol). Before the staining, a 3 h fixation in histofix (Carl Roth) and overnight incubation in PBS+ at 4 °C were performed. Samples were incubated in 750 µL staining solution (1 µg/mL of BodiPY and Hoechst in PBS+) for 1 h in the dark. Finally, they were washed twice in PBS+ for 15 min.

### 5.15. Anti-Perilipin A Staining

Perilipin A was visualized in fixated and halved hydrogels (see 2.14). Samples were permeabilized for 30 min (0.1% Triton-X in PBS+), blocked for 60 min (30 mg/mL bovine serum albumin and 0.1% Triton-X in PBS+), and washed three times for 15 min (0.1% Tween20 in PBS+). The primary rabbit anti-human perilipin A antibody (1:500, Sigma Aldrich) was incubated for 1 h, followed by washing steps. Incubation with goat anti-rabbit IgG-Alexa488 antibody (1:500, abcam) for 30 min in the dark followed. Samples were washed, and nuclei were stained with DAPI (1:1000, Serva) for 10 min in the dark.

### 5.16. Image Quantification

For semi-quantitative determination of intracellular lipid-positive and univacuolar-positive cells, ten representative image cutouts (1/4 of size) were counted.

### 5.17. Statistics

Data are obtained from three or five independent experiments. All values are shown as mean ± standard deviation. Significances were determined by one-way analysis of variance (ANOVA), followed by a post-hoc test (Tukey) or Kruskal-Wallis test using GraphPad Prism 9.3.1. Results were considered statistically significant when * *p* ≤ 0.05; ** *p* ≤ 0.01; *** *p* ≤ 0.001.

## Data Availability

The data presented in this study are available on request from the corresponding author.

## Supplementary Materials

The following are available online at https://www.mdpi.com/article/10.3390/gels8070420/s1, Figure S1: live-dead staining of encapsulated diffASCs: live-dead staining of diffASC in GG-hydrogels on days 56 and 84, viable cells in green, dead cells in red, and nuclei in blue. Scale bar 100 µM, n = 5.
