# Supplementary material for: Gellan Gum Is a Suitable Biomaterial for Manual and Bioprinted Setup of Long-Term Stable, Functional 3D-Adipose Tissue Models
Source: Gels. 2022 Jul 5;8(7):420. doi: 10.3390/gels8070420 (PMC9315477; doi:10.3390/gels8070420)
Supplement: Supplementary file 1 [file gels-08-00420-s001.zip › gels-1779271-supplementary.pdf]

**Supplementary Figure S1. Live-dead staining of encapsulated diffASCs:**

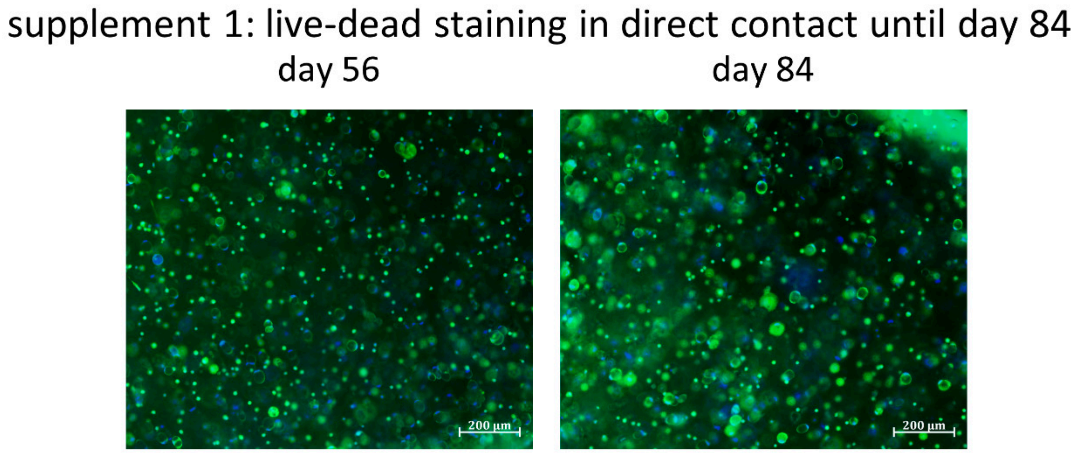


**Figure S1.** Live-dead staining of diffASC in GG-hydrogels on days 56 and 84, viable cells in green, dead cells in red, and nuclei in blue. Scale bar 100 µM, n = 5.
